# Accuracy of Self-Reported Sleep Position in Late Pregnancy

**DOI:** 10.1371/journal.pone.0115760

**Published:** 2014-12-23

**Authors:** Jane Warland, Jillian Dorrian

**Affiliations:** 1 School of Nursing and Midwifery, University of South Australia, Adelaide, Australia; 2 School of Psychology, Social Work and Social Policy, University of South Australia, Adelaide, Australia; 3 Centre for Sleep Research, University of South Australia, Adelaide, Australia; University of Alabama at Birmingham, United States of America

## Abstract

**Background:**

There is emerging research to suggest that supine maternal sleep position in late pregnancy may adversely affect fetal wellbeing. However, these studies have all been based on maternal report of sleeping position. Before recommendations to change sleep position can be made it is important to determine the validity of these studies by investigating how accurate pregnant women are in reporting their sleep position. If avoiding the supine sleeping position reduces risk of poor pregnancy outcome, it is also important to know how well women can comply with the instruction to avoid this position and sleep on their left.

**Method:**

Thirty women in late pregnancy participated in a three-night observational study and were asked to report their sleeping position. This was compared to sleep position as recorded by a night capable video recording. The participants were instructed to settle to sleep on their left side and if they woke overnight to settle back to sleep on their left.

**Results:**

There was a moderate correlation between reported and video-determined left-side sleep time (r = 0.48), mean difference = 3 min (SD = 3.5 h). Participants spent an average of 59.60% (SD = 16.73%) of time in bed on their left side (ICC across multiple nights = 0.67). Those who included left side among their typical sleep positions reported significantly longer sleep during the study (p<0.01).

**Conclusions:**

On average participant reports of sleep position were relatively accurate but there were large individual differences in reporting accuracy and in objectively-determined time on left side. Night-to-night consistency was substantial. For those who do not ordinarily sleep on that side, asking participants to sleep on their left may result in reduced sleep duration. This is an important consideration during a sleep-critical time such as late pregnancy.

## Introduction

There are a number of health care settings where it is important for the care provider to gather information about the client's typical and recent sleeping position. These include conditions involving muscular pain, heart burn, respiratory issues and commonly, obstructive sleep apnoea [1]. In those circumstances, changes to sleeping position can exacerbate or ameliorate client health. Health care professionals need to have confidence in client ability to self-report sleeping position and in many cases, the ability to comply with instructions to change sleep habits, in order to maximise positive health outcomes. There is increasing evidence that this is the case for pregnant women. To date, however, neither the accuracy of self-reported sleep position nor the ability to comply with instructions to change sleep position, have been studied in pregnancy. The importance in pregnancy is multiplied, with the possible health impact extending not only to the mother, but also the fetus.

It has been well understood that when a woman lies on her back during late pregnancy that the weight of the gravid uterus may cause inferior vena caval compression [2] and/or supine hypotension [3]. It is therefore established obstetric practice to move the pregnant woman to a left lateral position to aid recovery of the fetus during an episode of fetal bradycardia in labor [4], [5] or following an epidural [6]. Similarly, a lateral tilt is typically employed for any lengthy obstetric procedure such as during a caesarean section [7]. Interestingly, while care is taken to avoid women in late pregnancy remaining supine for any period of time, either at home or in the clinical context, comparatively little attention has been paid to time spent supine during sleep. In the last 12 weeks of pregnancy there is the potential for women to spend approximately 130 hours sleeping supine. This estimate is based on a recent study in 51 pregnant women, which found a median proportion of 26.5% of time asleep was spent supine, and an average nightly sleep time of 5.8 h [8].

In 2011, a study from New Zealand suggested that maternal supine sleep position may increase the odds of stillbirth (adjusted OR for supine sleep = 2.54, 95% CI 1.04 to 6.18) [9]. This triggered high-profile commentary, including an editorial in the British Medical Journal asking whether or not pregnant women should sleep on their left [10]. Indeed, the idea that guiding pregnant women to sleep on their left side could reduce the high rates of stillbirth, responsible for three million deaths worldwide each year [11], is powerful. The following year the ‘Sydney stillbirth study’ recruited 295 from eight Australian maternity hospitals and recently reported a six times increased risk in incidence of stillbirth in women who reported sleeping on their back [12]. Another cohort study reported in 2013 also supported these studies, finding increased odds of stillbirth (OR, 8.0; 95% CI, 1.5–43.2; P = 0.016) as well as low birth weight (OR, 5.0; 95% CI, 1.2–20.2; P = 0.025) associated with maternal supine sleep [13]. However, a major limitation with each of these studies, is that they relied on self-reported sleep position.

Two more studies currently underway, one in NZ [14] and the other in the UK [15], are also exploring the possible association between sleep position and poor pregnancy outcome. However, these two studies are also using self-reported sleep position. It is therefore of pressing importance to determine if pregnant women can accurately report their sleep position.

Understanding the accuracy of self-reported sleep position in this population is important in both the clinical, and the broader research context. Given that sleep becomes more difficult in late pregnancy [16], it is essential to determine the extent to which avoiding supine sleep and sleeping on the left is possible, particularly for those who do not include the left side among their typical sleep positions.

The aim of this study was to investigate these issues in a cohort of women during the last trimester of pregnancy. Our research questions were:

1. How accurate is self-reported sleep position in late pregnancy?
2. What happens to the sleep position and sleep time of women in late pregnancy when they are instructed to try to sleep on their left side?
3. How consistent is this across multiple nights?

## Materials and Methods

Thirty women in their third trimester (32–37 weeks) of an uncomplicated singleton pregnancy were recruited. The primary aim of the study was to investigate the relationship between reported left side sleep and objectively determined time on left. With thirty participants, we were sufficiently powered to detect a low to moderate relationship between these variables [critical r = 0.30, 1-ß = 0.80, α = 0.05].

### Ethics Statement

Approval from the University of South Australia's institutional research ethics committee (HREC) was gained for this study. Women who met the inclusion criteria were given a patient information sheet meeting the usual ethical requirements. They were assured that their personal information would remain anonymous and confidential, what to do if they changed their mind and who to contact if they required further information. After reading this, if they agreed to participate in the study they signed a consent form. In addition the woman's partner also gave written consent on the understanding that the video recording may include the partner, but that no information gained from this footage would be used in any way.

### Recruitment

Recruitment occurred through placing study flyers in community locations, such as pre-schools, schools and universities. Recruitment also was achieved via ‘snowball sampling’ i.e. women who had already participated in the study recruiting their pregnant family and friends.

All participants were aged 16 years and over, the age of consent for medical procedures in Australia. They were all literate in English because all study materials were in English. Women were excluded from the study if they had a booking (first visit in early pregnancy) BMI outside the normal-overweight range (18.5–29.9 kg/m2). This was to avoid known complications of obesity on sleep, such as sleep disordered breathing. They were also excluded if the pregnancy was complicated by medical or obstetric risks.

Participants were instructed to settle to sleep on their left side and if they awoke overnight to settle back to sleep on their left. They kept a night-time sleep diary for the three nights of the study. As each night progressed they were asked to keep this diary close to their bed so as to enable them to contemporaneously record their settling position, waking position and re-settling sleep position. Each morning participants were also asked to estimate the time they spent sleeping on their left and to document this information in their sleep diary. During the three nights of the study each participant was filmed as they slept via night-capable (Infra-Red (IR)) camera. Participants had complete control over the camera and could turn it on and off as they chose, however, only two women turned it off for a short period overnight. The remainder left it recording all night. Demographic information such as age, parity and current gestation was also collected as well as information regarding usual sleeping positions when not pregnant, (What positions do you usually sleep in when you are not pregnant? Please circle: Left, Right, Back, Tummy, Other), recent sleeping positions (What positions have you usually been sleeping in for the last month? Please circle: Left, Right, Back, Tummy, Other) the side of the bed the woman slept on, and any use of pillows.

### Data Analysis

The thirty women participated for three nights, yielding a total of 90 nights of data collection. Video recordings were incomplete or difficult to interpret for six nights, and on one of those nights, a participant did not complete their sleep diary. Therefore, the final dataset included 84 nights.

Multiple nights were recorded in order to maximise the likelihood that sleep would not be substantially affected by participants' knowledge of the presence of the video camera, and to allow the possibility of excluding the first night from analysis if a ‘first night effect’ [17] was apparent. In order to decide whether or not to include data from the first night of the study participants were asked to report if the camera had impacted on their sleep. None of the participants reported that the camera was so invasive that they would not be willing to do the study again (I would be happy to participate again: strongly agree = 27%; agree = 60%; neutral = 7%; missing = 7%). While mean subjective total sleep time (mean±SD; night1 = 7.2±1.2 h; night2 = 7.6±1.1 h; night3 = 7.5±1.2 h) and subjective sleep quality (mean±SD; night1 = 5.4±1.8; night2 = 6.1±1.4; night3 = 6.2±1.4 h), indicated that sleep was a little shorter and of lower quality on night 1, mixed effects regression models (random effect = subject ID, repeated across nights) indicated that there were no significant differences between nights (sleep time F_2,53.5_ = 1.7,p>0.05; quality F_2,52.3_ = 1.7,p>0.05). Nor were there any significant differences in subjective total sleep time (camera impact: yes = 7.5±1.1 h; no = 7.3±1.5 h) or subjective sleep quality (yes = 6.1±1.8; no = 5.6±1.5) between those who felt the camera had an impact and those who did not (sleep time F_1,75.1_ = 0.9,p>0.05; quality F_1,76.0_ = 1.0,p>0.05). Therefore, the first night of recording was included in the analyses.

Differences between participants across nights are illustrated in figures and an intraclass correlation coefficient (ICC) calculated to quantify intra-individual consistency. Differences in sleep and position measures (dependent variables) according to demographic variables (independent variables) were explored using linear mixed effects models with a random effect of subject ID. These models allowed individuals to vary in both intercept and slope, accounting appropriately for inter-individual differences and serial correlation across repeated observations [18].

## Results

### Demographics

The majority of participants were 26–30 years of age had completed post-secondary school education, and were having their first baby. There was a relatively even spread across care provider options. More than half (57%) of participants usually slept on the right hand side of the bed (from the perspective of the person in the bed) and most used another pillow, other than one under their head, in bed. Further demographics are displayed in Table 1.

**Table 1:** Sample demographics and typical sleep (n = 30).

variable	level	%
age group	16–25y	27%
	26–30y	47%
	31–40y	26%
education	Secondary school	13%
	post-secondary (not at a university, e.g. apprenticeship, TAFE)	27%
	additional study university	60%
care provider	obstetrician/consultant	37%
	midwife	37%
	shared care (GP plus hospital)	24%
parity	primiparous	70%
	multiparous	30%
I find sleeping on my left side comfortable	strongly agree/agree	53%
	neutral	27%
	disagree	20%
side of the bed I usually sleep on	left	37%
	right	57%
	single bed	7%
I sleep with a pillow	under head	100%
	between knees	43%
	bump	23%
	behind back	13%
	“pregnancy” pillow	17%

### Self-Report versus Video-Determined Time on Left

**Figure 1:**
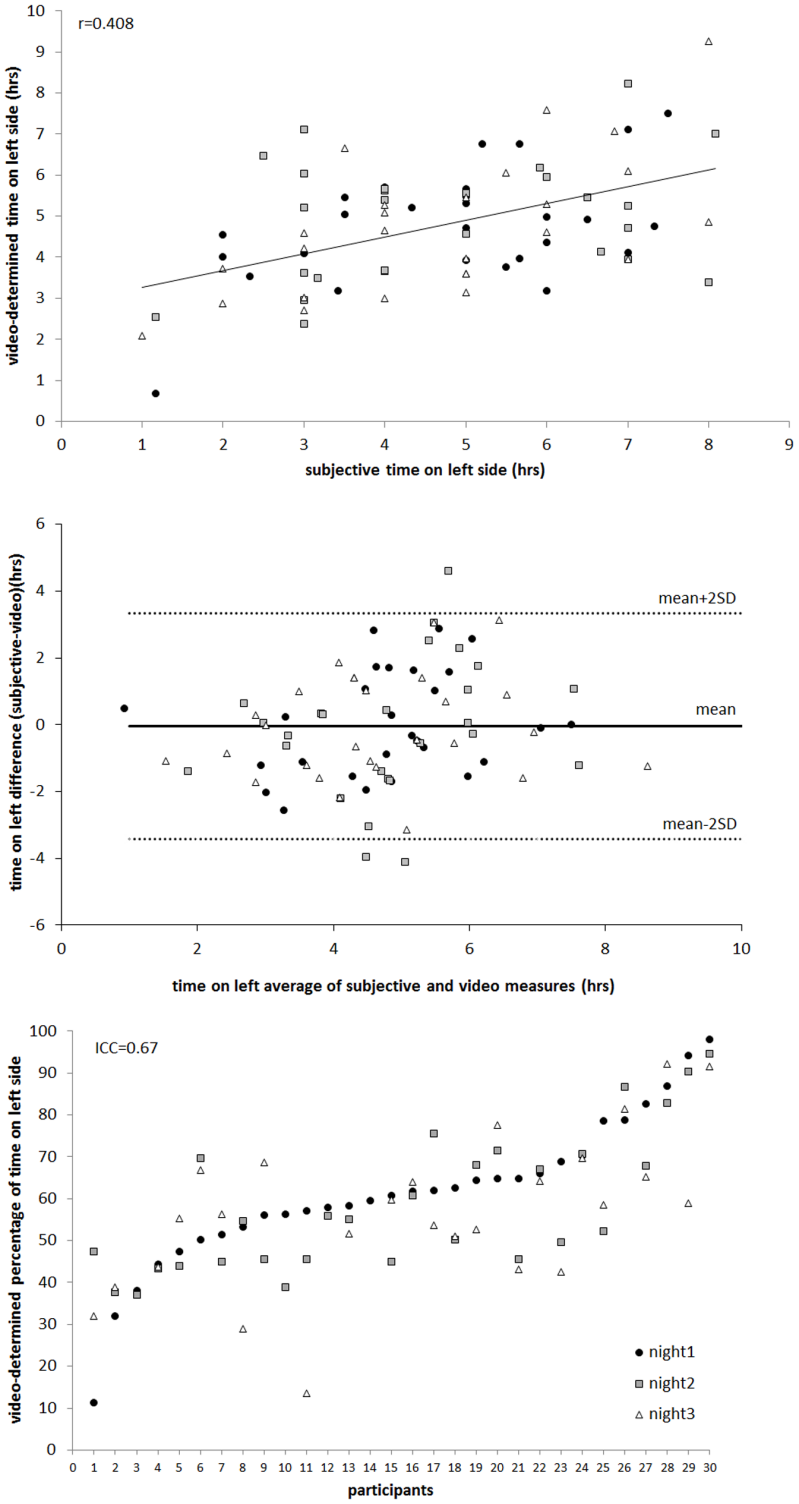
Upper Panel: Scatterplot plus line of best fit for subjective time on left side (x-axis) against video-determined time on left side (y-axis). Middle Panel: Bland-Altman Plot illustrating the relationship between subjective and video measures of time spent on left side. Night1 = filled circles, night2 = grey squares and night3 = grey triangles. Lower Panel: Video-determined percentage of time on left with participants on the x-axis sorted by night1 percentage from smallest to largest.

As illustrated in Fig. 1, upper panel, a basic correlation (not accounting for multiple observations per person) was moderate (r = 0.418, 23% of the variance). As displayed on the Bland Altman Plot (Fig. 1, middle panel), there was a mean difference of only 3 minutes between diary- and video-determined time on left side. However, there was a high degree of variability of nearly three and a half hours, as indicated by the mean±2*SD range lines.

### Typical Sleep during the previous month

**Figure 2:**
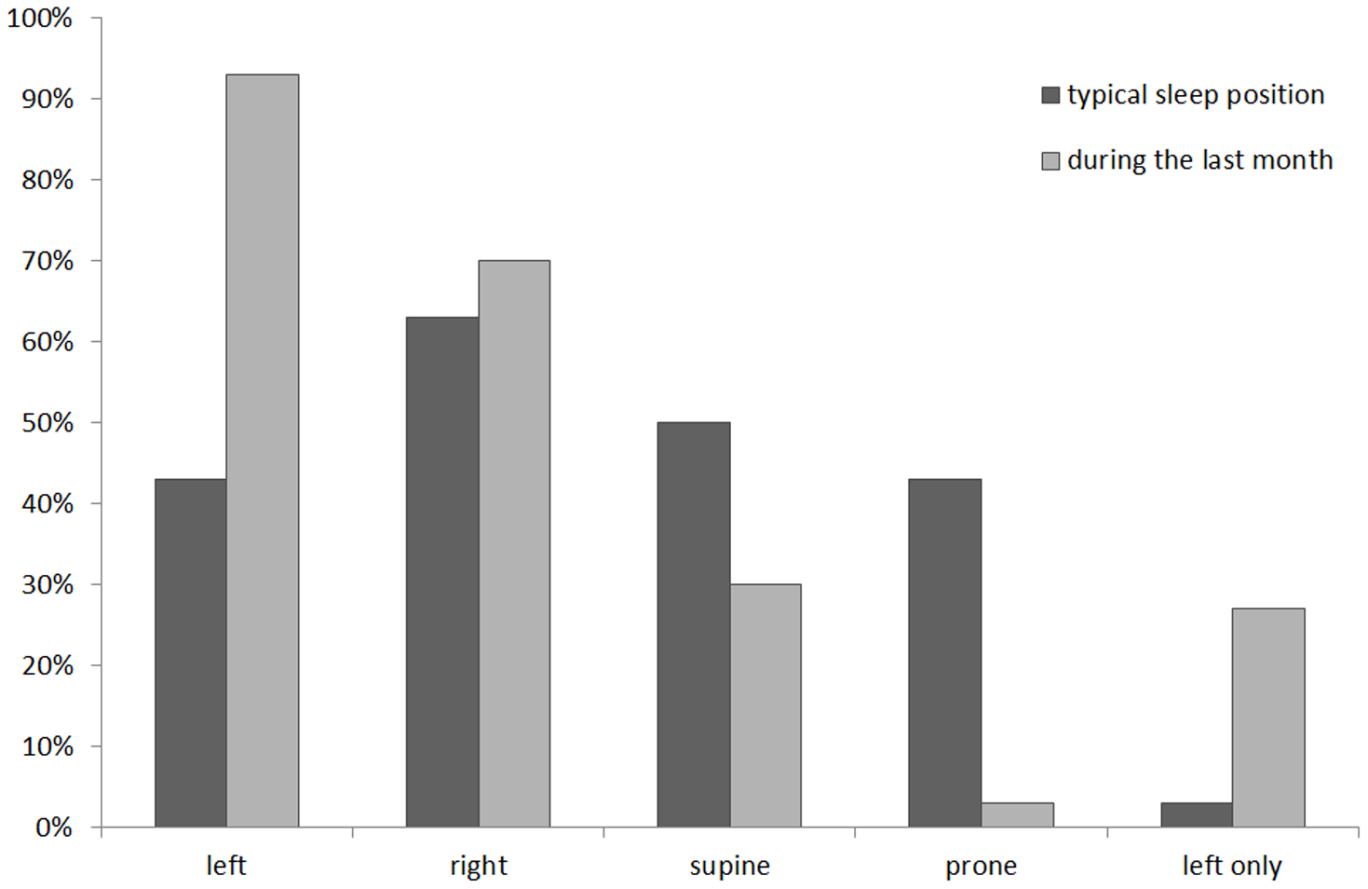
Proportion of participants including left, right, supine or prone among their typical sleep positions (prior to pregnancy), and among their sleep positions during the past month. Those reporting that they sleep on their left side only are reported on the far right.

While less than half (43%) of the participants reported typical positions that included the left side while not pregnant, during pregnancy (specifically in the month leading up to study participation), this increased to more than 90%. Similarly, while only one person typically slept exclusively on their left side, this increased to 27% during their previous month of pregnancy. The proportion of participants reporting sleep on their right side also increased during the preceding month and conversely reported supine and prone sleep reduced (Fig. 2).

### Description of sleep during the study

Average subjective sleep time during the study was 7.45 h (SD = 1.19 h), with 4.72 h (SD = 1.78 h) reported by participants as time on their left side. Average subjective sleep quality was 5.87 (SD = 1.75), around the middle of the 10-point scale. Video analysis indicated that participants spent an average of 7.99 h (SD = 1.34 h) in bed, with 4.77 h (SD = 1.50 h) of this time on the left side. An average of 1.91 h (SD = 1.27 h) was spent on their right side, 1.34 h (SD = 1.24 h) supine, and no time prone.

Video analysis indicated that during the study participants spent an average of 59.60% (SD = 16.73%) of time in bed on their left side. Fig. 1, lower panel, displays video-determined percentage of time on left (y-axis) for each participant (x-axis). Night1 (circles), 2 (squares) and 3 (triangles) are overlayed for each person and the figure is sorted by percentage of night 1 spent on the left side (left-right = smallest-largest). From the figure it is evident that time on the left was highly variable across participants, ranging from 11–98%. The figure also indicates a substantial level of intra-individual consistency (i.e. within individuals, across nights), as reflected in the ICC = 0.67.

### Differences in Sleep Time and Position

Mixed effects analyses are displayed in Table 2, which includes estimated marginal means and F-tests. Results of post-hoc comparisons for significant main effects are indicated in brackets following the in-text descriptions [for example (post hocs: a>b,c) would indicate that group a was significantly larger than group b and group c, but there were no significant differences between b and c]. Analyses indicated significant (p<0.05) differences in subjective sleep time by age (post hocs: 26–30y>31–40y,16–25y), education (secondary>additional study, university), parity (primipara>multipara) and whether or not participants reported that sleeping on their left was comfortable (agree<neutral, disagree), side of bed (left<right), typical sleep left (yes>no) and reported sleep during the last month on the left side only (yes>no). There were no significant differences in subjective time on left side for any of these variables. There were significant (p<0.05) differences in subjective sleep quality by age (26–30y>31–40y,16–25y).

**Table 2:** Estimated marginal means with standard errors (sterr), F (df in subscript) from the mixed effects models for Diary Measures (left panel) and Video Measures (right panel) by each of the seven model predictors (rows).

		Diary Measures						Video Measures					
		Sleep Time		Left Time		Quality		Time in Bed		Left Time		Left Time%	
		mean	(sterr)	mean	(sterr)	mean	(sterr)	mean	(sterr)	mean	(sterr)	mean	(sterr)
**age**	16–25y	**7.12**	**(.29)**	4.45	(.71)	**5.42**	**(.51)**	7.76	(.50)	5.28	(.48)	67.85	(5.47)
	26–30y	**9.25**	**(.34)**	5.95	(.81)	**7.72**	**(.60)**	9.32	(.57)	5.94	(.55)	62.60	(6.29)
	31–40y	**8.23**	**(.30)**	4.91	(.73)	**5.97**	**(.54)**	8.84	(.52)	5.40	(.50)	60.20	(5.67)
	F_df_ =	_1,13.7_	10.67**	_2,16.7_	1.1	_2,14.1_	5.08*	_2,16.2_	1.91	_2,17.2_	0.55	_2,17.0_	0.48
**education**	secondary	**9.81**	**(.47)**	6.90	(1.12)	7.65	(.83)	**10.00**	**(.79)**	**7.29**	**(.77)**	73.61	(8.72)
	additional	**7.60**	**(.25)**	4.31	(.61)	5.90	(.45)	**7.64**	**(.43)**	**4.23**	**(.42)**	55.84	(4.71)
	university	**7.20**	**(.25)**	4.10	(.61)	5.56	(.44)	**8.28**	**(.43)**	**5.11**	**(.42)**	61.20	(4.72)
	F_df_ =	_2,12.7_	11.49**	_2,16.4_	2.41	_2,13.9_	2.33	_2,15.5_	3.67**	_2,16.8_	6.64*	_2,16.4_	1.76
**parity**	primipara	**8.63**	**(.28)**	4.91	(.68)	6.51	(.49)	9.19	(.48)	5.66	(.46)	60.75	(5.25)
	multipara	**7.77**	**(.25)**	5.30	(.59)	6.23	(.45)	8.09	(.42)	5.43	(.41)	66.35	(4.61)
	F_df_ =	_1,13.1_	5.97**	_1,16.5_	0.21	_1,14.4_	0.21	_1,15.2_	3.42	_1,16.9_	0.16	_1,16.4_	0.73
**left comfort**	agree	**7.59**	**(.20)**	5.66	(.47)	5.99	(.35)	7.95	(.33)	5.44	(.32)	**69.41**	**(3.67)**
	neutral	**8.38**	**(.29)**	4.57	(.71)	6.96	(.53)	8.96	(.50)	4.86	(.49)	**52.45**	**(5.51)**
	disagree	**8.63**	**(.37)**	5.07	(.90)	6.16	(.65)	9.01	(.63)	6.32	(.62)	**68.80**	**(6.98)**
	F_df_ =	_2,12.6_	4.05*	_2,16.5_	0.84	_2,13.6_	1.37	_2,15.5_	1.81	_2,16.8_	2.5	_2,16.5_	4.13*
**side of bed**	left	**8.66**	**(.26)**	5.19	(.63)	6.84	(.46)	8.73	(.45)	5.92	(.43)	66.91	(4.91)
	right	**7.75**	**(.21)**	5.02	(.50)	5.90	(.38)	8.55	(.35)	5.16	(.34)	60.20	(3.90)
	F_df_ =	_1,13.0_	11.90**	_1,16.7_	0.07	_1,14.0_	4.02	_1,15.7_	0.16	_1,17.1_	3.17	_1,16.9_	1.88
**typical left**	yes	**8.65**	**(.29)**	4.85	(.70)	6.66	(.52)	9.10	(.50)	5.50	(.48)	58.07	(5.47)
	no	**7.75**	**(.22)**	5.36	(.53)	6.08	(.39)	8.18	(.37)	5.58	(.36)	69.03	(4.08)
	F_df_ =	_1,12.3_	7.50*	_1,16.2_	0.41	_1,13.4_	0.99	_1,15.0_	2.66	_1,16.6_	0.02	_1,16.3_	3.12
**left only**	yes	**8.76**	**(.33)**	5.59	(.80)	6.59	(.60)	**9.47**	**(.57)**	**6.41**	**(.55)**	69.01	(6.26)
	no	**7.64**	**(.19)**	4.61	(.46)	6.15	(.35)	**7.81**	**(.33)**	**4.67**	**(.32)**	58.09	(3.57)
	F_df_ =	_1,13.3_	8.79*	_1,16.8_	1.19	_1,14.4_	0.43	_1,16.0_	6.74*	_1,17.4_	7.89*	_1,17.1_	2.43

Where models revealed significant differences (p<0.05), means (sterr) are highlighted by bold boxes.

**p<0.01; *p<0.05.

There were significant (p<0.05) differences in video-determined time in bed by education (secondary>additional study, university) and reported sleep during the last month on the left side only (yes>no). There were also significant (p<0.05) differences in video-determined time on left side by education (secondary>additional study, university) and reported sleep during the last month on the left side only (yes>no). There were significant differences in video-determined percentage of time on left side by whether or not participants reported that sleeping on their left was comfortable (neutral<agree, disagree).

## Discussion

In answer to our research question as to whether or not pregnant women can accurately self-report their sleeping position, this study yielded a moderate correlation between diary-reported and video-determined indicators with an average difference of only three minutes between these measures. This supports the only other published study in the adult population which also suggested adults are reliable when reporting their sleep position [1]. Furthermore, this study lends support to the validity of findings from the earlier stillbirth studies which were based on self-report [9], [12]–[13]. However, given the wide individual differences in accuracy (up to three and a half hours) and the importance of the accuracy of self-reported sleep position, future prospective studies which further explore the possible association between maternal sleep position and poor pregnancy outcome would benefit from supplementing maternal report with an objective measure such as IR camera or a body position sensor.

As to the second study question concerning ability to comply with the instruction to sleep on their left side, video analysis indicated that participants spent around 60% of the night in this position. However, this was highly variable across participants, ranging from 11% to 98% (no one spent the entire night sleeping on their left side). In relation to the third study question around consistency, the percentage of time spent on left was relatively consistent within participants across multiple nights (ICC = 0.67). However, while the average difference in time spent on left between study nights was only fifteen percent, the maximum difference was more than forty percent. This suggests that for some people there may be large differences in sleep position from night to night, even under conditions where they are instructed to settle on their left. This study also found that those women who did not typically sleep on their left side achieved less sleep when asked to adopt this position. Disturbed sleep is common in pregnancy, however, it may be associated with a range of poor pregnancy outcomes such as gestational hypertension, gestational diabetes, preterm birth and depression [19]–[27]. Therefore, if sleep position is found to be causally associated with poor pregnancy outcome it would be important to determine ways to enable women who do not typically sleep on their left side to successfully change their sleeping position in pregnancy, whilst avoiding any negative consequence of disrupting their sleep. Strategies such as the use of pillows may need to be explored to facilitate comfort in this position. Other options to avoid sleeping on the back, such as the ‘tennis ball’ strategy, used in sleep disordered breathing research [28], may also be adapted and used in this context.

In an apparent paradox women who most strongly agreed that sleeping on their left side was comfortable spent less time asleep that the other participants. We hypothesise that this may be because this group of women were more confident that they could comply with the instruction to sleep on their left side for the entire night and this effort ultimately resulted in them becoming uncomfortable in maintaining this position, thereby missing out on sleep time. Consistent with this, those women who agreed and those that disagreed that sleeping on their left side was comfortable spent significantly more time on their left during the study than those who indicated that they held a “neutral” view. Alternatively this group may have gained a more restful sound sleep in the position in which they were most comfortable and therefore needed less time asleep. This is also a finding which would need to be further explored in future studies.

Curiously, those women who slept on the left side of the bed (from the perspective of the person in bed) achieved more sleep during the study. It may be that it is more comfortable to face out of the bed while sleeping than to face towards the bed partner. This would be interesting to study further as this suggests that women who sleep on the left side of the bed may be better able to maintain a left lateral sleeping position.

Even before their participation in our study, participants told us they had already increased the amount of time that they spent sleeping on their left at the end of their pregnancy compared with the way they slept prior to becoming pregnant. It may be that they are hearing or reading a “sleep on the left in pregnancy” message (e.g. [29]–[31]). It could be that they are naturally assuming this position [32], particularly as the option of prone sleep becomes more and more difficult as the pregnancy progresses. There is certainly scope for further research to explore reasons for this change.

### Limitations

Participants were asked to keep a sleep diary, which meant that all women were highly aware of their sleep position. Nevertheless, as sleep is something which takes place every night it may be that most people are in fact already aware of the position in which they usually sleep.

Due to the nature of this study it was not possible to record the amount of time women slept on their left side prior to our instruction to sleep on this side. Without this it is not possible to accurately gauge whether or not the women in our study actually increased the overall percentage of the night spent sleeping on their left side above pre-pregnancy levels. A recent study described typical sleep positions in late pregnancy (after 28 weeks) in 33 women in who were not instructed to adopt any particular position during sleep. Participants spent an average of 35% of the night in the left lateral position [8]. Taken together with results of the current study, these findings suggest that instructing women to sleep on their left side probably improves the amount of time spent in this position.

Beyond our basic self-reported sleep measures, it would have been beneficial to measure sleep time and architecture using polysomnography [PSG]. Given the potential negative impact of the electrodes on participants' comfort during the study, we opted instead for video monitoring. While this tells us about the time spent in bed and the time in each position, with a much lower participant burden than PSG, objective measures of sleep in future studies would allow greater understanding of the impact of position on sleep quality, especially if those with shorter sleep duration had higher quality [less disturbed] sleep.

## Conclusions

Emerging research suggests that if pregnant women avoid sleeping on their back that this may be an important step in reducing incidence of poor pregnancy outcomes such as stillbirth. This study shows that asking women to spend more time sleeping on their left may be feasible. While on average, women accurately reported their left lateral sleep time, the wide individual differences highlight that when speaking with women on an individual level, the differences between their perception of left side sleeping time and the actual time spent on the left side may be as large as three and a half hours.

Whilst bearing in mind that asking any person, pregnant or not, to remain in one position all night is practically difficult [33] and understanding that none of the participants actually spent all of the night in this position, it will be important for future research to determine how much left-sided sleeping time is needed in order for any protective effect to occur. Such research could explore the interaction between sleep position and stage of sleep, whether sleep position during daytime napping is important, the interplay between other factors such as maternal weight, presence or absence of sleep disordered breathing, as well as maternal blood pressure and gestational age. Further research may also be able to identify populations for whom left side sleeping may provide a substantive decrease in risk, compared with other “low risk” populations, where left sleeping may not provide much advantage.
